# N-terminal acetylation and replicative age affect proteasome localization and cell fitness during aging

**DOI:** 10.1242/jcs.157354

**Published:** 2015-01-01

**Authors:** Sjoerd van Deventer, Victoria Menendez-Benito, Fred van Leeuwen, Jacques Neefjes

**Affiliations:** 1 Division of Cell Biology, Netherlands Cancer Institute and Netherlands Proteomics Center, Plesmanlaan 121, 1066CX Amsterdam, The Netherlands; 2 Division of Gene Regulation, Netherlands Cancer Institute and Netherlands Proteomics Center, Plesmanlaan 121, 1066CX Amsterdam, The Netherlands

**Keywords:** Proteasome, Intracellular location, N-acetylation, Replicative age, Aging

## Abstract

Specific degradation of proteins is essential for virtually all cellular processes and is carried out predominantly by the proteasome. The proteasome is important for clearance of damaged cellular proteins. Damaged proteins accumulate over time and excess damaged proteins can aggregate and induce the death of old cells. In yeast, the localization of the proteasome changes dramatically during aging, possibly in response to altered proteasome activity requirements. We followed two key parameters of this process: the distribution of proteasomes in nuclear and cytosolic compartments, and the formation of cytoplasmic aggregate-like structures called proteasome storage granules (PSGs). Whereas replicative young cells efficiently relocalized proteasomes from the nucleus to the cytoplasm and formed PSGs, replicative old cells were less efficient in relocalizing the proteasome and had less PSGs. By using a microscopy-based genome-wide screen, we identified genetic factors involved in these processes. Both relocalization of the proteasome and PSG formation were affected by two of the three N-acetylation complexes. These N-acetylation complexes also had different effects on the longevity of cells, indicating that each N-acetylation complex has different roles in proteasome location and aging.

## INTRODUCTION

The proteasome is a major intracellular protease and controls many processes, including protein quality control. Protein quality control is required to prevent accumulation of damaged proteins during the lifespan of a cell (Amm et al., 2014; Koga et al., 2011). Insufficient recognition and clearance of damaged proteins can yield harmful protein aggregates (Powers et al., 2009; Schmidt and Finley, 2014). A proper functioning ubiquitin-proteasome system (UPS) might prevent protein aggregation and counteract cellular aging.

Several studies report an age-dependent decline in UPS activity in various model systems (Carrard et al., 2002; Dasuri et al., 2009; Lee et al., 1999; Vernace et al., 2007a; Vernace et al., 2007b). Other studies suggest a causative relation between UPS activity and aging. Enhancing proteasome activity by overexpression of the proteasome assembly chaperone Ump1 improves budding yeast longevity under starvation conditions (Chen et al., 2006). Increasing proteasome levels by overexpressing Rpn4, a protein which drives the transcription of the proteasome subunits, also increases the replicative lifespan in *S. cerevisiae* (Kruegel et al., 2011). These studies suggest that the UPS system decays with age and limits the lifespan of cells and organisms. Manipulating UPS therefore might have dramatic effects on the aging process.

For several reasons, *S. cerevisiae* is an important model organism to elucidate the molecular basis of processes related to aging. First, cell division is asymmetrical with a distinguishable mother and daughter cell. This allows tracking of a single cell over time, even during division. Second, the number of cell divisions can be quantified by counting the bud scars left on the mother cell after budding of a new generation. The asymmetrical cell division defines two forms of aging; chronological aging and replicative aging (Kaeberlein, 2010; Michal Jazwinski et al., 1989). Chronological aging is defined as the time between the budding from the mother, the birth, until the daughter cell dies. This aging is usually addressed on a population level by measuring the viability of a liquid culture upon starvation (Kaeberlein, 2010). Replicative aging is aging as a result of cell division and defined by the number of daughter cells produced by an individual mother cell. Replicative aging in yeast is used to model aging of mitotically active mammalian cells (Kaeberlein, 2010; Mortimer and Johnston, 1959). Chronological and replicative aging are overlapping processes (Delaney et al., 2013; Kennedy et al., 1994; Murakami et al., 2012), exemplified by the observation that, during starvation of a liquid yeast culture, the replicative age of a cell at the start of starvation highly affects the chronological age that will be reached (Allen et al., 2006; Aragon et al., 2008). The studies in yeast have revealed many insights into the various molecular processes underlying aging and is expected to provide handles to manipulate aging related diseases such as neurodegenerative disorders (Clay and Barral, 2013; Tenreiro and Outeiro, 2010).

Here, we followed two proteasome-related processes that occur during chronological aging in yeast: nuclear-cytoplasmic relocalization of proteasomes, and the formation of cytoplasmic proteasome storage granules (PSGs). PSGs are aggregate-like structures that contain the proteasome and form early during yeast starvation (Laporte et al., 2008). The replicative age of cells had a major effect on these processes. Replicative young cells efficiently relocalized the proteasome from the nucleus and formed PSGs, unlike replicative old cells. A genome-wide knockout screen revealed that proteasome relocalization and PSG formation involves two of the three N-acetylation complexes, each having a particular effect on proteasome localization. The N-acetylation complexes were found to affect cell fitness in different ways. One N-acetylation complex, NatC, both affected proteasome location and fitness of old cells.

## RESULTS

### Proteasome localization during starvation correlates with replicative age

Proteasomes equally distribute over the nucleus and cytoplasm in mammalian cells (Reits et al., 1997). In the budding yeast *Saccharomyces cerevisiae*, proteasomes accumulate in the nucleus when cells have sufficient nutrients (Russell et al., 1999). This changes when yeast cells exhaust glucose in the growth medium, a process that leads to starvation. During starvation, cells relocalize their proteasome from the nucleus into the cytosol and form cytoplasmic PSGs (Laporte et al., 2008). Starvation is apparently a factor controlling the intracellular distribution of proteasomes.

**Fig. 1.**
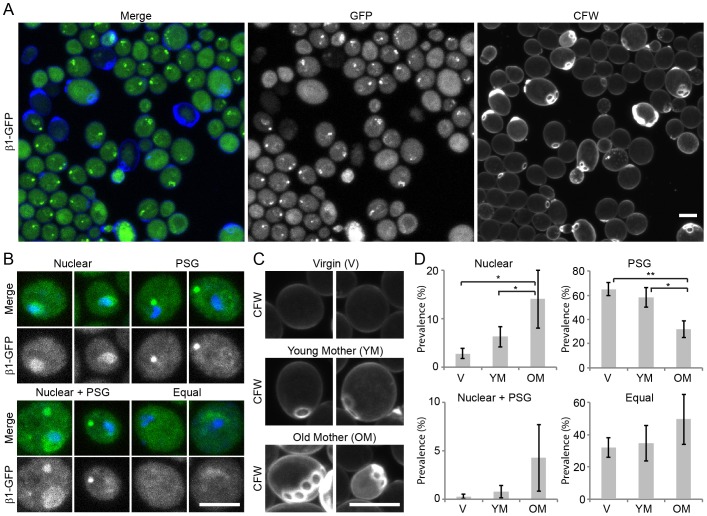
**Proteasome localization in nutrient-starved cells correlates with replicative age.** (A) Live-cell microscopy of yeast cells in starvation shows various 20S proteasome localizations as is visualized by endogenous expression of a GFP-tagged β1 subunit (Pre3). Cells were stained with CFW to assess the replicative age of individual cells. (B) β1–GFP localization and Hoechst 33342 staining was used to define four different phenotypes: cells with cytosolic PSGs (PSG), cells with nuclear enrichment of proteasomes (Nuclear), cells that display both a nuclear enrichment of proteasomes and PSGs (Nuclear + PSG), and cells without a clear enrichment of proteasomes in PSGs or nuclei (Equal). (C) Based on CFW staining of bud scars, three different replicative age groups were defined: virgin daughter cells without bud scars (V); young mother cells with 1–2 bud scars (YM); and old mother cells with more than two bud scars (OM). (D) The prevalence of the different proteasome phenotypes in living cells from each age group was calculated by dividing the number of cells with a certain phenotype in a particular age group over the total number of cells in this age group. Results are mean±s.d. based on three independent experiments. Significance was calculated with a paired, two-tailed Student's *t*-test (*P<0.05, **P<0.01). Scale bars: 5 µm.

We visualized proteasomes in live yeast cells by tagging the catalytically active β1 subunit (Pre3) of the proteasome with GFP. Efficient and quantitative introduction of the β1–GFP in 20S proteasomes was confirmed by native gel electrophoresis (supplementary material Fig. S1). The GFP-labeled proteasomes had a similar distribution during starvation as reported for non-modified proteasomes previously (Laporte et al., 2008). We observed that cells in starvation show a wide heterogeneity in proteasome localization (Fig. 1A). Based on proteasome localization, we defined four localization phenotypes: (1) cells with proteasome accumulation in the nucleus (Nuclear); (2) cells displaying dots of cytoplasmic proteasome clusters (PSG); (3) cells displaying both PSGs and a nuclear accumulation of proteasomes (Nuclear + PSG); and (4) cells without any of these phenotypes, where proteasomes are approximately equally distributed between the cytoplasmic and nuclear compartments (Equal) (Fig. 1B). In a typical 5-day starvation experiment the majority of the cells are either PSG or Equal, whereas a small portion of the cells is Nuclear or Nuclear + PSG.

Given that these cells are genetically identical and grow under identical conditions, it is expected that other factors should be responsible for this heterogeneity. These could include replicative aging. Replicative aging results from asymmetrical cell division of budding yeasts in which damaged cell components are typically retained in the mother cell (Kaeberlein, 2010). After each cell division, chitinous scar tissue is left on the cell wall of the mother, which is called a bud scar. The number of bud scars can be visualized with Calcofluor White (CFW), which marks the replicative age (Pringle, 1991). CFW staining distinguishes three age groups: (1) virgin cells without bud scars; (2) young mother cells with one or two bud scars, and (3) old mothers with more than two bud scars (Fig. 1C). We quantified the respective proteasome localization phenotypes per age group. PSG formation inversely related to age as ∼30% of the old mothers, ∼60% of the young mothers and ∼65% of the virgin cells displayed this phenotype (Fig. 1D). Nuclear accumulation of proteasomes correlated with replicative age in the small population of yeast where this phenotype was observed. This suggests that impairment of proteasome relocalization and/or PSG formation can be associated with replicative age. The prevalence of the other two phenotypes did not differ significantly between the different age groups. Similar results were found in yeasts expressing proteasomes labeled through another 20S subunit [α2­–GFP (Pre8)] or a 19S subunit (Rpn1–GFP) (supplementary material Fig. S2).

### Identification of genes affecting proteasome localization during starvation

To identify genetic factors controlling proteasome localization during starvation, we performed a microscopy-based yeast knockout screen. We considered two explanations for the maintenance of nuclear enrichment of the proteasome during starvation: altered proteasome biogenesis in the nucleus or altered nuclear retention of the proteasome. Therefore, we tagged the β1 subunit (Pre3) of the proteasome with a fluorescent recombination induced tag exchange (RITE) cassette (Verzijlbergen et al., 2010), to differentially label new and old proteasomes. Integration of the RITE cassette behind the β1 gene results in a GFP-tagged proteasome produced before tag exchange, whereas new proteasomes [produced after tag exchange due to translocation of an estrogen receptor (ER)-coupled Cre-recombinase to the nucleus after addition of β-estradiol (Verzijlbergen et al., 2010)] will be labeled with mRFP. The genetic GFP-for-mRFP swapping is permanent and induced after two days of starvation. When recombination was induced at this time point little or no synthesis of proteasomes was detected in wild-type (WT) cells. Similar results have been obtained by Menendez-Benito et al. for several other proteins in these starvation conditions (Menendez-Benito et al., 2013).

**Fig. 2.**
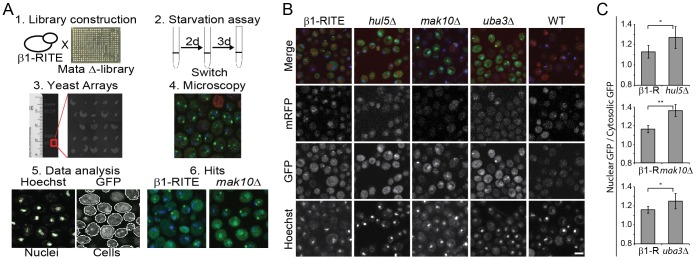
**A genome-wide screen identifying genes affecting nuclear proteasome localization during starvation.** (A) Schematic overview of the screening. (1) A yeast knockout library was crossed with a β1–GFP→mRFP RITE strain. (2) Tag recombination (switch) was performed after 2 days during a 5-day starvation experiment. (3) Samples were fixed, stained with Hoechst 33342 and printed on yeast arrays. (4) Microscopic imaging of GFP (old proteasomes), RFP (new proteasomes) and Hoechst 33342 (nuclei) was performed. (5) Images were analyzed by CellProfiler. (6) *mak10Δ* was one of the hits for a nuclear proteasome enrichment. (B) Confocal microscopy images of three hits showing nuclear enrichment of GFP-labeled proteasome in the nucleus: *hul5Δ*, *mak10Δ* and *uba3Δ*. Only background signal is observed for the mRFP proteasome. (C) Quantification of nuclear:cytosolic ratios of GFP in WT and nuclear retention hits. Results are mean±s.d. based on five independent experiments. Significance was calculated with a paired, two tailed Student's *t*-test (*P<0.05, **P<0.01). Scale bar: 5 µm.

To obtain a screening library, the β1–RITE strain was crossed with the MAT**a** haploid knockout (KO) collection (Thermo Scientific) using SGA technology (Tong et al., 2001) (Fig. 2A1). This high-throughput crossing yielded 4263 knockout strains containing a RITE-tagged proteasome. These strains were subjected to a 5-day starvation protocol, including the induction of tag exchange (switch) at day 2 (Fig. 2A2). To efficiently analyze thousands of samples by microscopy, cells were fixed, stained with Hoechst 33342 and spotted on an object glass using a DNA microarray printer (Narayanaswamy et al., 2006) (Fig. 2A3). Each spot, typically consisting of ∼2000 cells, was imaged by confocal microscopy (Fig. 2A4). A CellProfiler image analysis pipeline was designed for quantification of the proteasome phenotypes of interest (Carpenter et al., 2006) (Fig. 2A5). This pipeline assessed the nuclear cytosolic distribution of the proteasome by dividing the mean GFP fluorescence in the nucleus over the mean GFP fluorescence in the cytoplasm. Three successive rounds of screening identified three hits with a nuclear retention phenotype of the proteasome: *hul5Δ*, *uba3Δ* and *mak10Δ* (Fig. 2A6; Fig. 2B). These results were verified by repeating the experiment with independently made knockout strains. Loss of *HUL5*, *UBA3* or *MAK10* increased the population of cells with nuclear accumulation of proteasomes (Fig. 2C). Little or no synthesis of new (mRFP tagged) β1 was detected in either WT or KO cells, thus implying that the nuclear enrichment is not due to *de novo* synthesis. A plating assay before and after recombination confirmed the successful genetic recombination (GFP to mRFP) in these cells (supplementary material Fig. S3A). When recombination is induced at an earlier time point in starvation (after 1 day), synthesis of new (mRFP tagged) proteasomes could be observed in both WT cells and the three screen hits (supplementary material Fig. S3B). The RITE technology was only used for identifying the hits.

### Loss of N-acetylation by NatC causes nuclear enrichment of the proteasome without affecting PSG formation, and both phenotypes are affected by replicative age

**Fig. 3.**
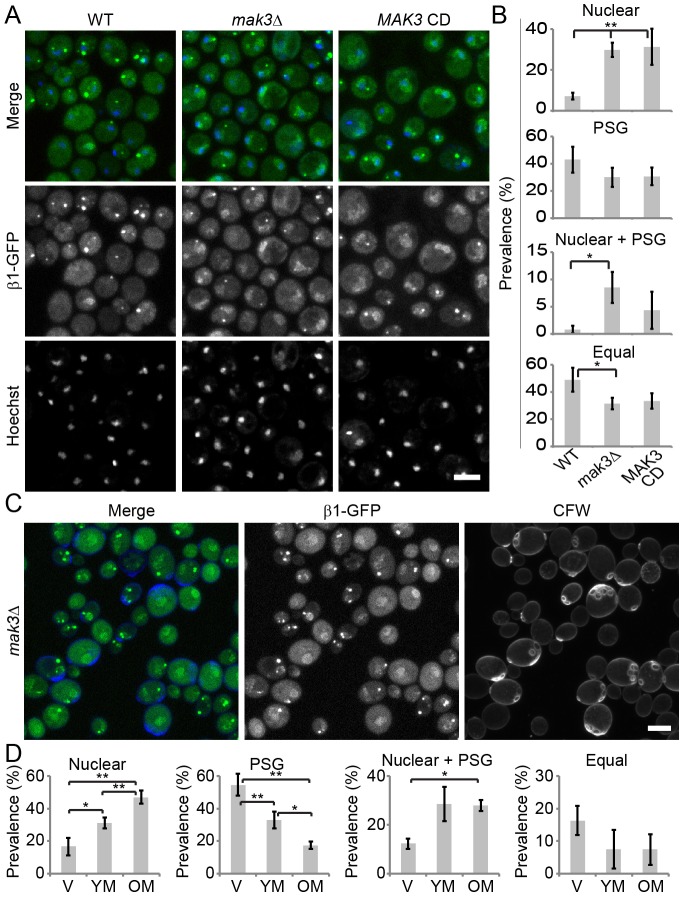
**Loss of N-acetylation by NatC causes nuclear retention of the proteasome without affecting PSG formation with both phenotypes being affected by replicative age.** (A) Fixed-cell microscopy of Hoechst-33342-stained *mak3*Δ cells or cells expressing a catalytically inactive Mak3 (MAK3-CD) showing an increased population of cells displaying nuclear retention of proteasomes after a 5-day starvation period. (B) The prevalence of the different phenotypes in the total population was scored in three independent experiments. (C) Live-cell microscopy of *mak3Δ* cells stained with CFW after a 5-day starvation period. (D) The prevalence of each proteasome phenotype in living cells was scored in the three different age groups (V, virgin daughter cells; YM, young mother cells; OM, old mother cells). Results are mean±s.d. based on three independent experiments. Significance was calculated with a paired two-tailed Student's *t*-tests (*P<0.05, **P<0.01). Scale bars: 5 µm.

The Mak10 protein is a subunit of the N-acetyltransferase C (NatC) complex. NatC associates to the ribosome for co-translational N-terminal acetylation of a subset of proteins (Starheim et al., 2012). The NatC complex further consists of Mak31 and the catalytic subunit Mak3 (Polevoda and Sherman, 2000). In our screening, Mak3 and Mak31 were just below the threshold, but independently generated knockouts of all three individual NatC subunits showed increased nuclear retention of proteasomes during starvation, whereas the number of cells displaying PSGs was not significantly altered (Fig. 3A,C; supplementary material Fig. S4A,B), indicating a specific role for the NatC complex in the nuclear enrichment of the proteasome. The presence of cells with both nuclear enrichment of proteasomes and cytoplasmic PSGs means that PSG formation has been uncoupled from the nuclear-to-cytosolic relocalization of proteasomes (Fig. 3A,B). A catalytically inactive NatC mutant (Mak3 N123A and Y130A) (Polevoda and Sherman, 2000) showed the same phenotype (Fig. 3A,B). NatC activity is apparently involved in the nuclear-to-cytosolic relocalization of proteasomes under starvation conditions. CFW staining was used to assess a potential correlation of proteasome localization with replicative age. Nuclear enrichment of proteasomes correlated with replicative age, whereas PSG formation correlated negatively with replicative age (Fig. 3C,D). Cells displaying both nuclear enrichment and PSG formation also showed a weak correlation, but ‘Equal’ cells did not. Similar results with respect to the correlation between replicative age and proteasome localization and the effect of NatC deficiency were found for α2–GFP- (Pre8) and Rpn1–GFP-expressing cells (supplementary material Fig. S2). These results are similar to those observed for WT cells (Fig. 1D), indicating that NatC does not affect aging related relocalization of proteasomes.

### Nuclear-to-cytosolic relocalization of the proteasome during starvation requires NatB and NatC, PSG formation requires only NatB

**Fig. 4.**
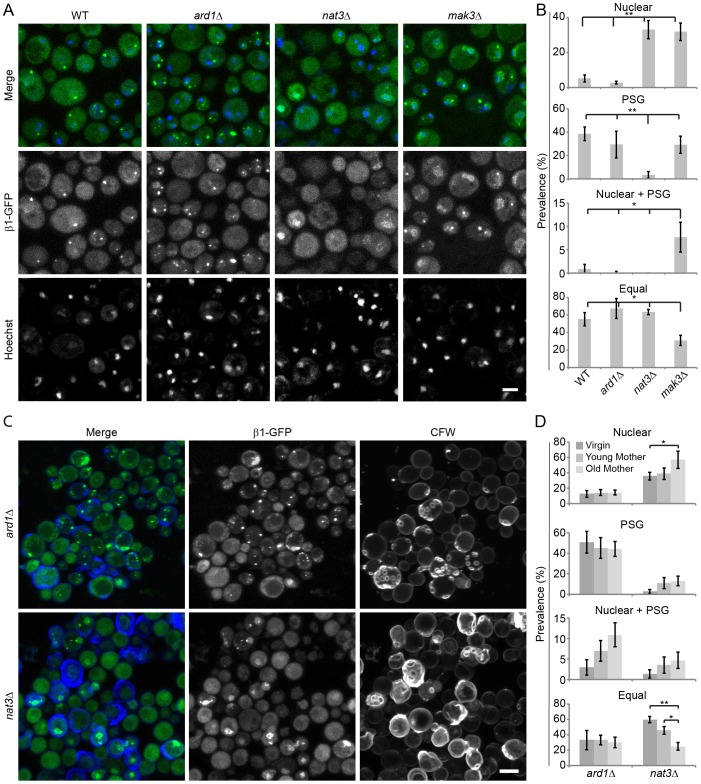
**Nuclear-to-cytosolic relocalization of the proteasome during starvation requires N-acetylation by NatB and NatC.** (A) Fixed-cell microscopy of starved *ard1*Δ (NatA deficient), *nat3*Δ (NatB deficient) and *mak3*Δ (NatC deficient) cells. Nuclei were visualized with a Hoechst 33342 staining. (B) Prevalence of the different proteasome localization phenotypes was scored in the total population. Results are mean±s.d. and are based on a biological triplicate. (C) Live-cell imaging of starved *ard1*Δ and *nat3*Δ cells. Cells were stained with CalcoFluor White to assess their replicative age. (D) Prevalence of the different proteasome phenotypes in the three age groups in living cells was quantified (mean±s.d.) in three independent experiments and significance was calculated with a paired two-tailed Student's *t*-test. (*P<0.05, **P<0.01). Scale bars: 5 µm.

The main N-terminal acetyltransferases in yeast are NatA, NatB and NatC, contributing to respectively ∼50%, ∼20% and ∼20% of the N-terminal acetylome. Each of these complexes recognizes specific substrates depending on their N-terminal sequences (Starheim et al., 2012). To test whether nuclear enrichment of proteasomes only depends on NatC-mediated N-acetylation, the subunits of the NatA (Ard1, Nat1 and Nat5) and the NatB (Nat3 and Mdm20) complex were knocked out. Deficiency of NatA subunits did not alter proteasome distribution following starvation (Fig. 4A,B;supplementary material Fig. S4C,D). However, deletion of the various NatB subunits increased the population with nuclear proteasomes and reduced the cells displaying PSGs (Fig. 4A,B; supplementary material Fig. S4E,F). Although both NatB and NatC knockouts induced nuclear proteasome enrichment, they had different effects on the cytoplasmic proteasome pool. NatC inactivation still allowed PSG formation, whereas NatB inactivation prevented formation of PSGs (Fig. 4A,B; supplementary material Fig. S4C–F). This suggests a specific role of the NatB complex in PSG formation. Cells with both PSGs and nuclear retention were hardly detected among NatB-knockout cells (Fig. 4B). As for WT and NatC-deficient cells, nuclear enrichment induced by NatB inactivation correlated with replicative age, whereas the prevalence of ‘Equal’ cells decreased with replicative age. (Fig. 4C,D; compare to Fig. 3D). Surprisingly, NatA-deficient cells did show a WT-like prevalence of the different proteasome phenotypes in the total population, but there was no correlation of these phenotypes with replicative age.

These results would suggest that the mechanism underlying the different proteasome localizations in cells involves selective N-acetylation and can be (at least partially) uncoupled from aging effects that also require N-acetylation. NatB and NatC, unlike NatA, are involved in the effects on proteasome distribution. Their combined inactivation might further accelerate these effects, and a NatB+NatC double knockout strain was made. This double knockout (unlike the single knockouts) had severe growth defects, preventing a fair comparison with the single knockout strains. Given that the affects on nuclear enrichment of the proteasome were specific to NatB and NatC, N-acetylation of one or more NatB and NatC substrates must be involved in nuclear-to-cytosolic proteasome distribution. Based on the N-terminal sequence requirements of each Nat complex a list of potential substrates were defined in the yeast proteome (Arnesen et al., 2009; Polevoda and Sherman, 2003). The role of N-acetylation of selected candidate substrates was tested by making an N-terminal MX- to MP- (X2P) mutation, resulting in an N-terminus that cannot be N-acetylated (Polevoda and Sherman, 2003). Preventing N-acetylation of α5 (Pup2), α6 (Pre5), Rpn9, Fub1, Avo2, Hul5 or Nup100 failed to phenotypically mimic cells lacking NatB or NatC (supplementary material Fig. S4G). Whether NatB and NatC act on proteasome distribution by modifying a single target or many, is as yet unclear.

### NatA and NatB control general cell fitness during starvation and NatC fitness of old cells only

**Fig. 5.**
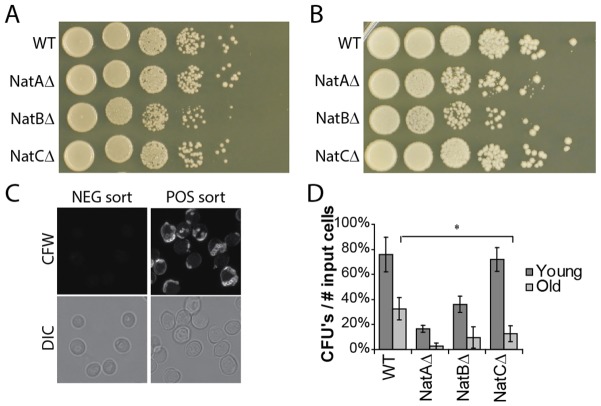
**In starvation, loss of NatA and NatB has a general effect on reproductive capacity, whereas loss of NatC specifically affects old cells.** The reproductive capacity of the different strains in log phase (A) and starvation (B) was assessed by a plating assay. Loss of NatA and NatB compromised reproductive capacity in starved cells, whereas loss of NatC did not. A representative plating assay from three independent experiments is shown. (C) After CFW staining of a starved culture, the cells with the lowest (negative) and highest (positive) CFW signal were sorted to obtain populations of virgin cells and old mothers, respectively. The CFW image is the maximum projection of a 5-µm *Z*-stack, the DIC image is a single scan in the middle of the *Z*-stacks. (D) CFUs were counted for old mother (old) and virgin (young) cells. Loss of NatC affects the reproductive capacity of old cells, whereas loss of NatA and NatB reduce the reproductive capacity of both young and old cells. Results are mean±s.d. based on on three independent experiments. Significance was calculated with a paired two-tailed Student's *t*-test. (* = *P*<0.05, ** = *P*<0.01).

As proteasome composition and activity was found to influence longevity in starvation, we wondered whether proteasome localization would also correlate with cell fitness. Cellular fitness in starvation can be determined by assaying the ability of cells to restart their cell cycle when nutrients are added. This is determined by plating equal numbers of cells and quantifying the number of colony-forming units (CFUs). When grown in the presence of sufficient nutrients, the reproductive capacity of NatA- and NatC-deficient cells is similar to WT cells, whereas NatB-deficient cells show a lower reproductive capacity (Fig. 5A) (Polevoda et al., 1999). After a 5-day starvation period, both NatA- and NatB-deficient cells showed lower CFUs than wild-type cells, whereas the reproductive capacity in NatC-deficient cells seemed to be unaffected (Fig. 5B). Proteasome localization in NatA-deficient cells was similar to WT, unlike that in NatB-deficient cells. Given that NatA and NatB deficiency both decrease the number of CFUs measured, proteasome localization cannot be directly related to reproductive capacity in starvation.

Given that NatC-deficient cells show a strong correlation of proteasome localization with replicative age, we wondered whether NatC also affected reproductive capacity in an age-dependent manner. We determined the fitness of old versus young cells in a starved population of the various mutant yeast strains by staining the yeast cells with CFW and then separating young and old yeast cells by FACS sorting (Pringle, 1991). Microscopy on the sorted populations verified separation of virgin and old mother cells (Fig. 5C). Equal numbers of cells from the different populations were subsequently plated and the number of CFUs determined. About 75% of the young WT cells and ∼30% of the old WT cells were able to form colonies upon plating (Fig. 5D). This correlation between replicative age and cell fitness is similar to results previously reported by Allen et al. (Allen et al., 2006). The low number of CFUs measured for old as well as young NatA- and NatB-deficient cells was expected, based on their general effects on cell fitness (Fig. 5B) and is in agreement with findings for NatA by Aragon et al. (Aragon et al., 2008). Surprisingly, WT and NatC-knockout young cells were equally fit, whereas the fitness of the old NatC-knockout cells was reduced to only 30% of old WT cells. NatC deficiency not only affected localization of proteasomes in replicative old cells, but it also had a selective effect on the fitness of old mothers.

## DISCUSSION

The proteasome is located in both the cytosol and nucleosol. Its subunits are made in the cytosol where proteasomes are assembled in precursor complexes that can be imported into the nucleus for full formation of the complex (Lehmann et al., 2002). Nuclear import and export of mature proteasomes is a very slow process in mammalian cells and the details of this process are poorly understood (Reits et al., 1997). Given that the nuclear envelope disintegrates during mitosis in mammalian cells, the boundary between the two pools of proteasomes is lost and nuclear proteasomes mix with cytosolic proteasomes. Nuclear proteasomes could thus have a cytosolic origin and vice versa. This should be different in budding yeast, where the nuclear envelope is maintained during cell division and proteasomes tend to accumulate in the nucleus. However, nuclear-to-cytosolic relocalization of proteasomes is observed upon glucose exhaustion, which is followed by rapid nuclear import of mature proteasomes upon re-addition of nutrients. It would therefore be expected that proteasome distribution is under some kind of control (Laporte et al., 2008).

We report that nuclear-to-cytosolic relocalization of the proteasome upon starvation correlates with the replicative age of yeast cells. Young cells are better capable of relocalizing the proteasome from the nucleus and of forming cytosolic PSGs, whereas old mother cells usually fail to do so. This correlation might be due to an age-dependent defect in the proteasome relocalization machinery, but could also be due to asymmetrical division of damaged cell components upon cell division. In general, damaged protein accumulates in mother cells leaving daughter cells with a fresh set of proteins (Henderson and Gottschling, 2008; Kaeberlein, 2010; Nyström and Liu, 2014). Perhaps a low amount of damaged proteins allows young cells to store their proteasomes in PSGs, whereas old cells might need to maintain a larger pool of active proteasomes to handle accumulated protein damage at the cost of PSGs. A nuclear enrichment of the proteasome might help replicative old cells to handle protein stress in the nucleus (Gardner et al., 2005) but it might also contribute to protein quality control in the cytosol (Heck et al., 2010; Prasad et al., 2010).

The reported association between the capacity of the ubiquitin proteasome system and viability during aging (Carrard et al., 2002; Kruegel et al., 2011; Tonoki et al., 2009; Vernace et al., 2007b) suggests that the localization of the proteasome also affects viability. This can only be tested in a system where proteasome localization can be manipulated during the aging process. We performed a microscopy-based screen to identify proteins that control proteasome localization in yeast cells. We identified two of the three complexes involved in N-terminal acetylation of proteins as controlling nuclear-to-cytosolic relocalization of the proteasome. About half of the yeast proteome can be N-acetylated by these complexes (Starheim et al., 2012). N-acetylation can both stabilize and de-stabilize proteins as well as affect the intracellular localization and activity of proteins (Arnesen, 2011; Hwang et al., 2010; Scott et al., 2011). Given that the acetylation complexes NatB and NatC but not NatA control the nuclear-to-cytosolic relocalization of proteasomes, a selective set of substrates rather than the general N-acetylation process seems to be responsible. The different phenotypes for the NatB and NatC knockouts allowed testing of the link between nuclear-to-cytosolic relocalization of proteasomes and the formation of PSGs. Although NatC-deficient old mother cells accumulated significantly more nuclear proteasomes, the prevalence of cytosolic PSGs was unaffected. In addition, a population of cells with both nuclear retention and PSG formation was observed. Both observations suggest that nuclear accumulation of proteasomes in older mothers does not necessarily prevent formation of cytosolic PSGs. This suggests that the mere enrichment of proteasomes in the cytosolic compartment is not a prerequisite for PSG formation.

As proteasome localization during aging can be manipulated by inactivation of either NatB or NatC, the effect on cell fitness during aging can be determined. Although NatA and NatB deficiency strongly affected cell fitness in all age groups tested, NatC deficiency selectively affected fitness of old mothers. As for the localization of the proteasome, the different Nat complexes affected cell fitness in a Nat-complex-specific manner. Whether this is the result of modification of part of the proteome or of one defined substrate, is at present unclear. We excluded some proteins that were potentially involved, like Hul5 (supplementary material Fig. S4G), another hit in our screening, as single candidates but that does not exclude other proteins.

Acetylation of lysine side chains of various proteins has been connected to the aging process. This is exemplified by the yeast deacetylase Sir2. Sir2 reduces lifespan upon deletion and prolongs it upon overexpression (Wierman and Smith, 2014). Homologs of Sir2 in several other organisms, including the SIRT proteins in mammals, have been linked to aging and age-related diseases (Donmez and Guarente, 2010). Furthermore, calorific restriction can increase age and has been associated with altered acetylation status of many mitochondrial proteins (Hebert et al., 2013), which has been extensively studied in neurodegeneration diseases (Guedes-Dias and Oliveira, 2013). These examples indicate a role of acetyl modifications of lysine side chains in processes associated with aging. Here, we have shown that N-terminal acetylation, a different, stable and often co-translational modification with the same chemical group, is associated with proteasome distribution and fitness during aging. The mechanisms controlling proteasome localization and fitness at old age involve specific N-acetylation complexes and result in a further expansion of the role of the small acetyl modification.

## MATERIALS AND METHODS

### Yeast strains and plasmids

With the exception of the strains used for screening, all strains were derived from NKI4103 (Verzijlbergen et al., 2010). Gene knockouts were made by PCR-mediated gene disruption based on pRS plasmids (Baker Brachmann et al., 1998). N-terminal mutations were made by PCR amplification of the pYM-N10 and pYM-N11 plasmids using the S1 and S4 primers extended with 40 bp of sequence homologous to the endogenous sequence (Janke et al., 2004). The N-terminal mutation was introduced in the 40 bp endogenous sequence. The two mutations for the catalytically inactive N123A-Y130A-Mak3 were generated by Delitto Perfetto technology (Storici and Resnick, 2006). All strains are described in supplementary material Table S1.

### Growth Conditions

Yeast cells were grown in liquid YEPD cultures of 5 ml at 30°C. To prevent recombination of the RITE cassette, cells were grown in presence of Hygromycin (200 µg/ml, Invitrogen). Liquid cultures were starved by inoculating 5 ml of YEPD with 0.5 ml of an overnight culture followed by a 5-day starvation period.

### Library construction

NKI4103 (Verzijlbergen et al., 2010) was crossed with the MAT**a** haploid knockout collection (ThermoScientific) by Synthetic Genetic Array analysis (Tong and Boone, 2006) using a RoToR HAD (Singer Instruments) with the following modifications. After mating, diploids were selected and kept on Hygromycin, G418 and CloNat triple selection on rich medium for 1 night. After 2 weeks on sporulation medium, MAT**a** haploid clones containing both a gene knockout and the RITE tagging system were selected. The first two rounds of selection generated haploid MAT**a** cells (YC-His+Can+SAEC) (van Leeuwen and Gottschling, 2002). The next two selection rounds selected the knockout and the RITE system (YC-His+Can+SAEC+MSG+ Hygromycin, G418 and CloNat).

### Microscopy and image analysis

Fixed microscopy samples were prepared by fixing ∼10^8^ cells in 4% formaldehyde and mounted in Vectashield (Vector Laboratories) on ConA-coated coverslips. Live-cell samples were prepared by resuspending ∼10^8^ cells in 100 µl 40°C 1% UltraPure™ LMP Agarose (Invitrogen) in PBS which is squeezed between a cover glass and an object glass. Imaging was performed at room temperature within 1 hour after mounting. Hoechst 33342 (Invitrogen, 1 µg/ml) or Calcofluor White (CFW, Sigma-Aldrich, 2 µg/ml) staining was performed before mounting the sample. Images were made on a Leica SP5 (Leica Microsystems), using a 63× objective and a 405-nm laser to excite Hoechst 33342 and CFW, a 488 nm laser for GFP and 561 nm for mRFP. 5 µm thick *Z*-stacks were made with 15 slides. Image analysis was performed on maximum Z-projections by scoring different phenotypes, counting 200–500 cells per biological replicate.

### Screening

The screening of the β1-RITE + KO library was performed in batches of 384 strains. A RoToR HAD (Singer Instruments) was used to transfer the strains from 384-well glycerol stocks to a YEPD+Hygromycin agar plate. 120 µl start cultures in 96-well plates were inoculated from the YEPD plate, grown overnight and used to start a 4 ml culture. A 2-day starvation period was followed with a switch assay as described by Verzijlbergen et al. (Verzijlbergen et al., 2010) and another 3-day starvation period. Samples were then fixed in 4% formaldehyde, stained with Hoechst and spotted on a yeast array (Narayanaswamy et al., 2006). Microscopic analysis was performed on a Leica AOBS LSCM (Leica Microsystems) using 405-, 488- and 561-nm laser light to excite Hoechst 33342, GFP and mRFP respectively. High-throughput image analysis was performed using CellProfiler software (Carpenter et al., 2006). The screening results of selected candidates were validated by two additional rounds of analysis and independent generation of the knockout yeast strains.

### Flow-based sorting of old and young cells

To isolate replicative old and young cells, ∼2×10^7^ cells of a starved culture were stained with CFW and FACS sorted on a MoFlo-Astrios (Beckman Coulter) using 405 nm excitation and collecting fluorescence emission with a 450 nm (30-nm bandpass) filter. The 2.5% of the cells with the highest and lowest CFW signal were isolated and ∼250 yeasts were subsequently plated on YEPD plates (Allen et al., 2006). CFUs were counted after 3 days culture at 30°C.

### Protein extraction and native gel analysis

Native protein samples were made by washing a cell pellet of ∼10^8^ cells in PBS plus protease inhibitors (1 mM PMSF, 5 mM benzamidine, 1 µg/ml pepstatin, 1 µg/ml leupepting) and resuspended in buffer A (20 mM Tris-HCl pH 7.4, 5 mM MgCl_2_, 1 mM DTT, 1 mM ATP) plus protease inhibitors. Cells were lysed in buffer A by using glass beads, which were removed before addition of a blue loading buffer (5×; 50% glycerol and Bromophenol Blue). Samples were loaded on a NativePAGE™ 3–12% Bis-Tris gel (Life Technologies) and ran in NativePAGE™ running buffer (Life Technologies). GFP fluorescence was visualized on a ProXPRESS (Perkin Elmer) machine with 480 nm (30-nm bandpass) excitation and 550 nm (40-nm bandpass) emission filters. To visualize untagged proteasomes, the gel was incubated with 100 µM suc-LLVY-AMC (Enzo Life Sciences) in the presence of 1 mM ATP, 1 mM DTT and 0.02% SDS (Elsasser et al., 2005). Gel scans were made with 390 nm (70-nm bandpass) excitation and 450 nm (20-nm bandpass) emission filters.

## Supplementary Material

Supplementary Material
