# Supplementary material for: N-terminal acetylation and replicative age affect proteasome localization and cell fitness during aging
Source: J Cell Sci. 2015 Jan 1;128(1):109–17. doi: 10.1242/jcs.157354 (PMC4282048; doi:10.1242/jcs.157354)
Supplement: Supplementary Material [file supp_128_1_109__index.html]

N-terminal acetylation and replicative age affect proteasome localization and cell fitness during aging — Supplementary Material 

# N-terminal acetylation and replicative age affect proteasome localization and cell fitness during aging

## JCS157354 Supplementary Material

**Files in this Data Supplement:**

- **Supplementary Material**
