# Supplementary material for: N-terminal acetylation and replicative age affect proteasome localization and cell fitness during aging
Source: J Cell Sci. 2015 Jan 1;128(1):109–17. doi: 10.1242/jcs.157354 (PMC4282048; doi:10.1242/jcs.157354)
Supplement: Supplementary Material [file supp_128.1.109_JCS157354.pdf]

Fig. S1

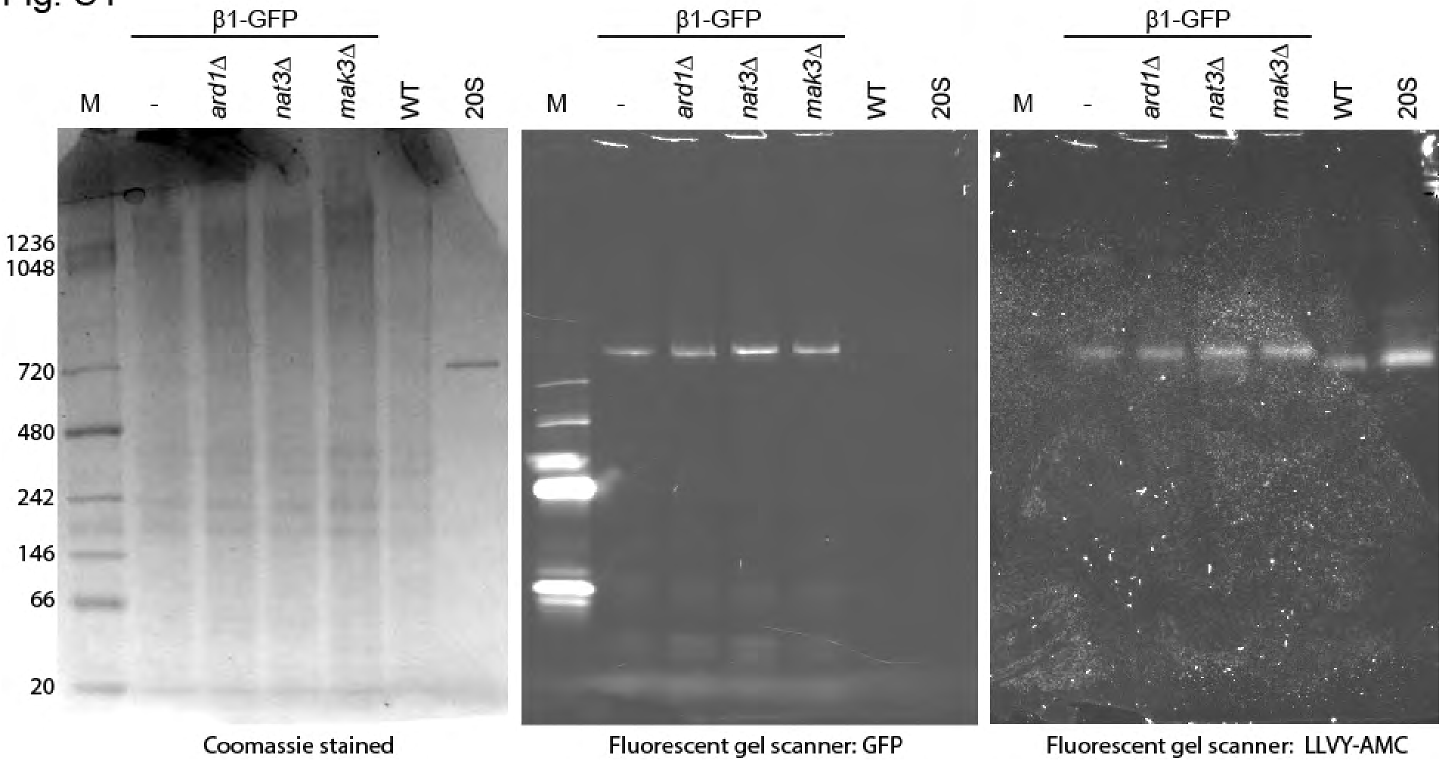

Fig S1: β1-GFP is efficiently incorporated in the 20S proteasome in WT and NatA, B or C deficient cells
A fluorescent gel scan of a native gel shows one GFP fluorescent band around 720 kDa for both β1-GFP and NatA, B or C deficient starved cells. This band runs at the same height as a purified 20S control (Mouse 20S proteasome, Boston Biochem) and is also present in a WT sample without GFP as was visualized by incubating the gel with a suc-LLVY-AMC (Enzo Life Sciences) proteasome activity probe. The marker (M) was visualized by Coomassie staining of the same gel. We conclude that the presence of the GFP tag on β1 doesn't induce the presence of proteasome assembly intermediates or a pool of unincorporated substrates in β1-GFP or NatA, B or C deficient cells.

Fig S2

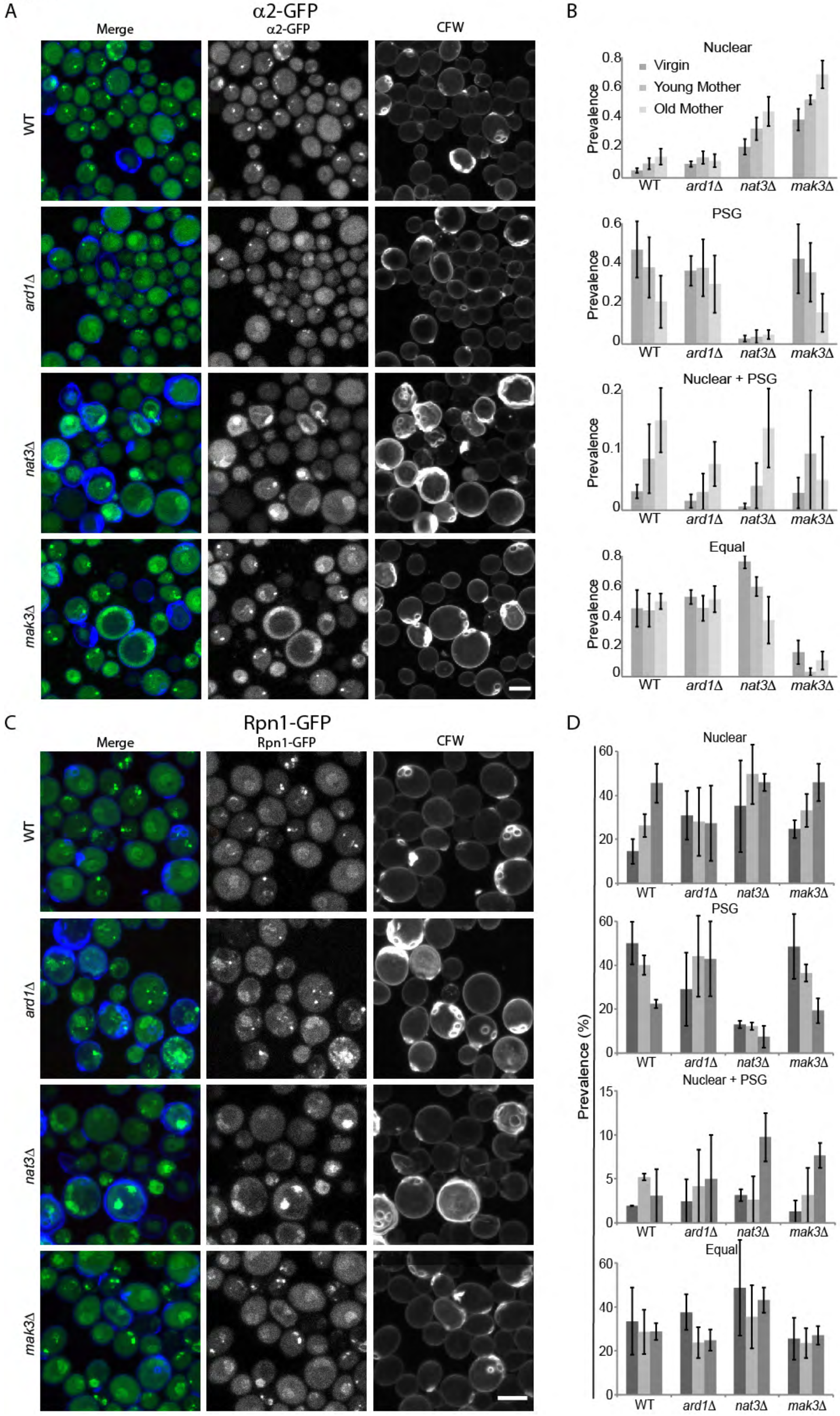
A
α2-GFP
Merge
α2-GFP
CFW
WT
ard1Δ
nat3Δ
mak3Δ
B
Nuclear
Virgin
Young Mother
Old Mother
Prevalence
0.8
0.6
0.4
0.2
0
PSG
Nuclear + PSG
Equal
C
Rpn1-GFP
Merge
Rpn1-GFP
CFW
D
Nuclear
PSG
Nuclear + PSG
Equal
Prevalence (%)

Fig S2: GFP tagged α2 (Pre8) and Rpn1 show the same proteasome localization as β1-GFP in WT and NatA,B or C deficient cells **(A)** Live cell microscopy of starved WT and NatA (*ard1*Δ*)*, NatB (*nat3* Δ*)* or NatC (*mak3* Δ) deficient cells endogenously expressing α2(Pre8)-GFP. Cells were stained with CalcoFluor White to asses replicative age. **(B)** The prevalence of the different phenotypes in the different age groups was scored in two independent experiments (~200 live cells per replicate). **(C)** The same experiment as in (A), but now with cells endogenously expressing RPN1-GFP, a component of the 19S base particle. **(D)** Prevalence of the different proteasome phenotypes in the three age groups of living cells. (Scale bars, 5 µm)

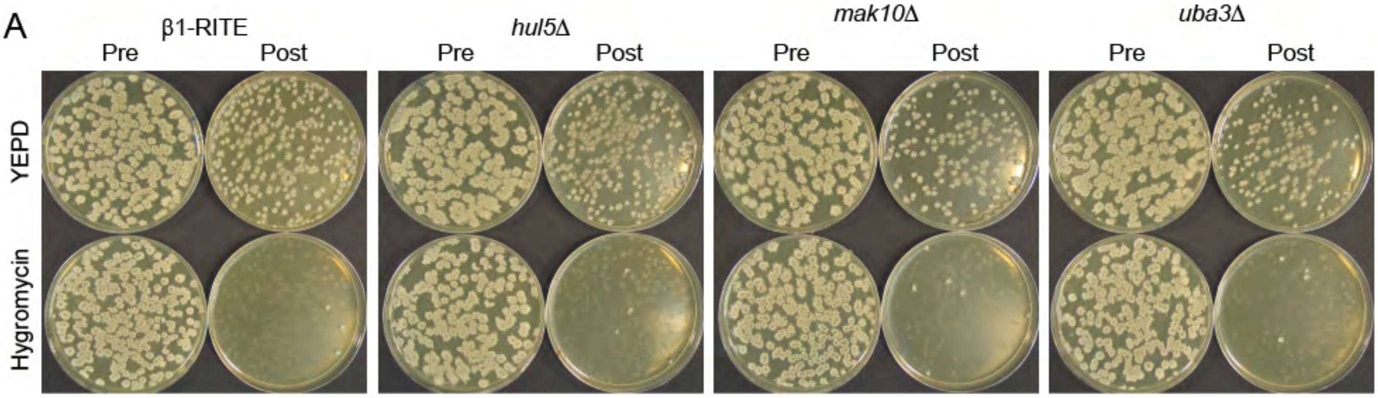

% recombined cells:

	β1-RITE	*hul5Δ*	*mak10Δ*	*uba3Δ*
Pre	2%	11%	2%	4%
Post	96%	97%	96%	96%

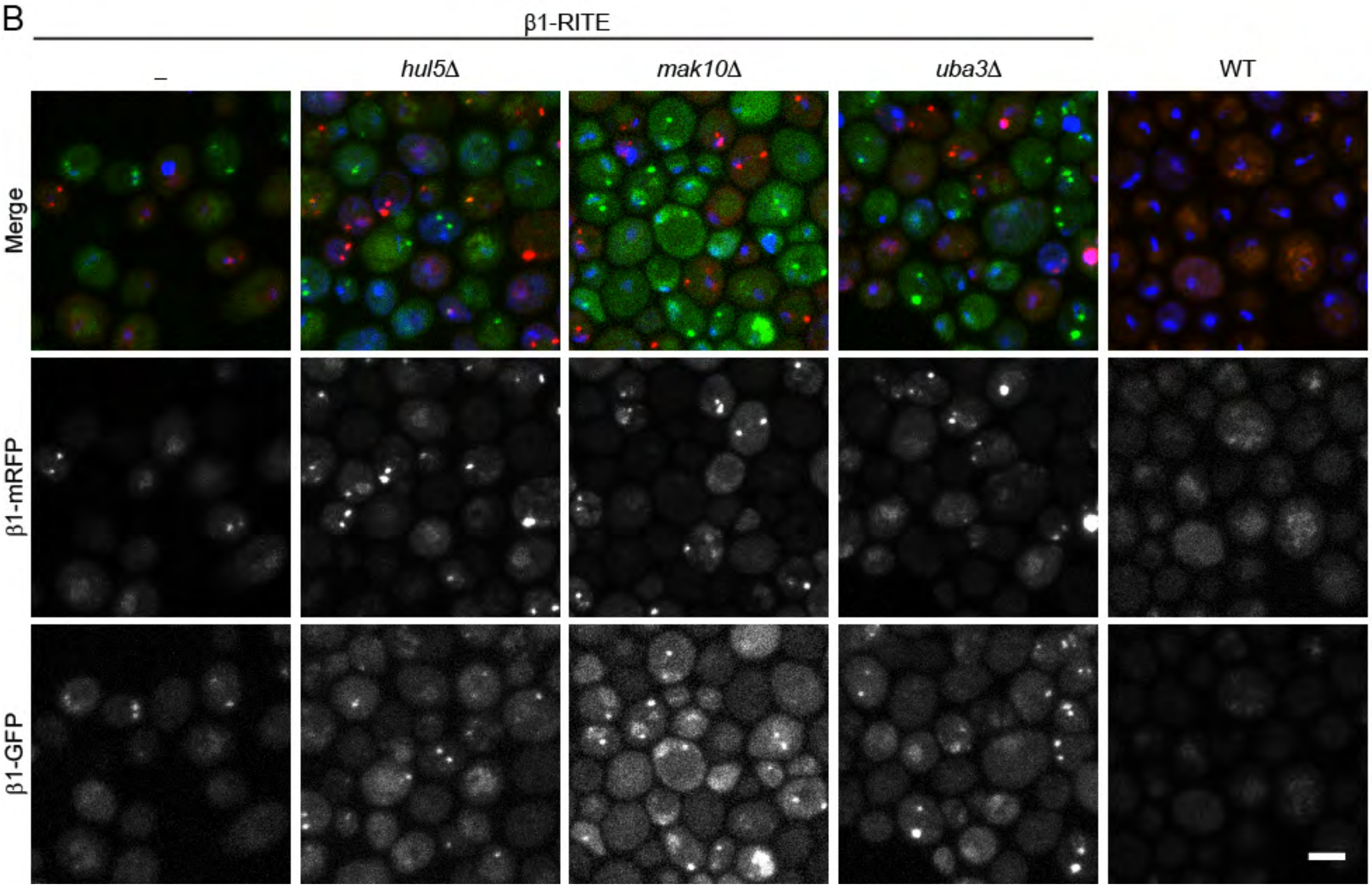

Fig S3: Plating assay confirms efficient recombination in *hul5Δ*, *mak10Δ* and *uba3Δ* cells
(A) Genetic recombination of the RITE cassette results in the loss of Hygromycin resistance, which can be used to assess the percentage of cells that underwent a recombination event as described by (Verzijlbergen et al., 2010). Samples were taken just before (Pre) and ~16h after (Post) recombination was induced. In both WT and mutant cells the recombination without induction is low and the induced recombination is high. **(B)** Live cell microscopy of WT cells and the three nuclear retention hits. Images were taken after 5 days starvation, but the recombination was induced after one day starvation instead of two days. Under those conditions synthesis of new (mRFP-tagged) proteasome can be detected, thereby validating the RITE tool in starvation conditions. (Scale bar, 5 μm)

Fig S4

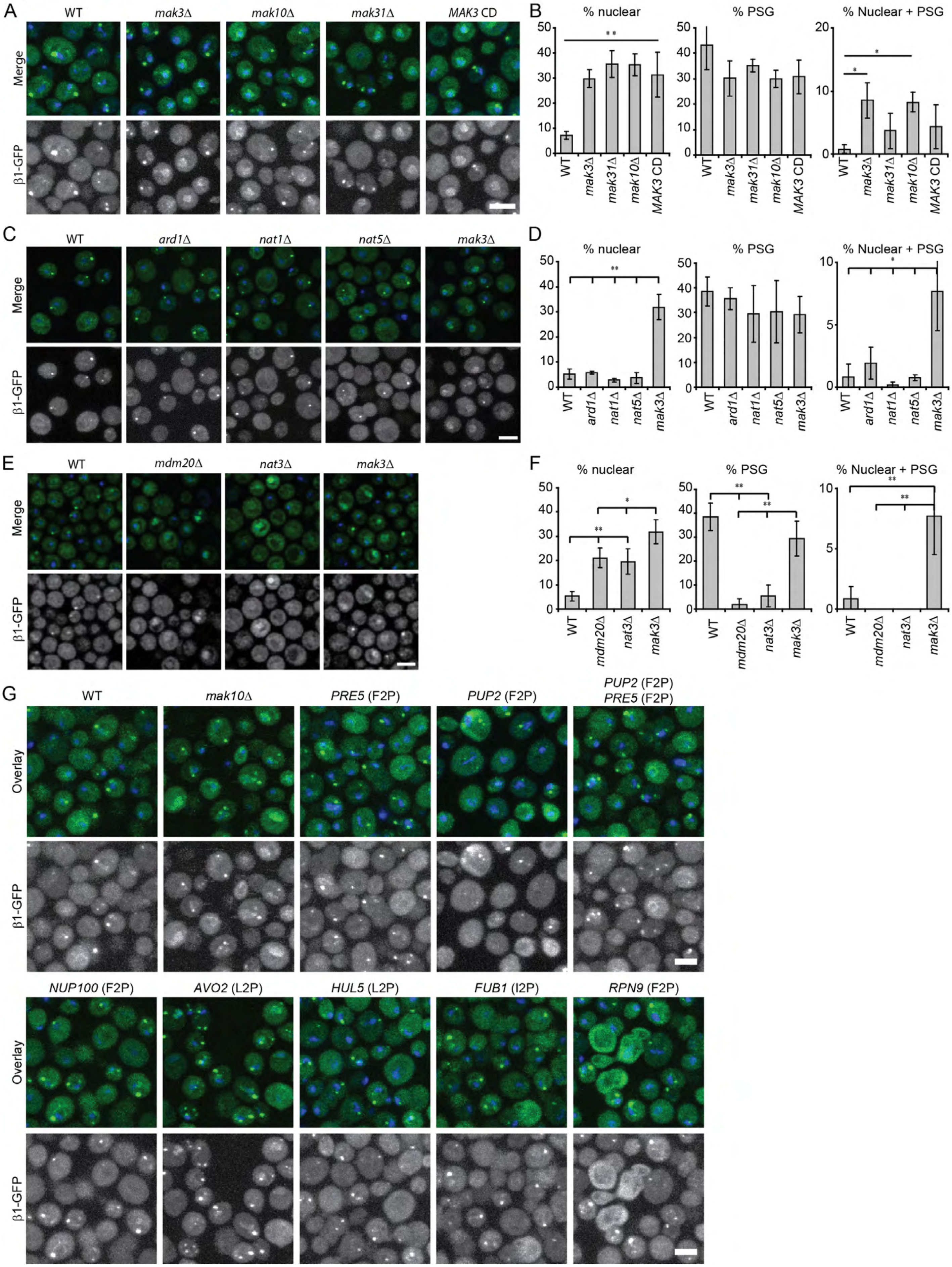
A
WT
mak3Δ
mak10Δ
mak31Δ
MAK3 CD
Merge
β1-GFP
B
% nuclear
% PSG
% Nuclear + PSG
WT
mak3Δ
mak31Δ
mak10Δ
MAK3 CD
C
WT
ard1Δ
nat1Δ
nat5Δ
mak3Δ
Merge
β1-GFP
D
% nuclear
% PSG
% Nuclear + PSG
E
WT
mdm20Δ
nat3Δ
mak3Δ
Merge
β1-GFP
F
% nuclear
% PSG
% Nuclear + PSG
G
WT
mak10Δ
PRE5 (F2P)
PUP2 (F2P)
PUP2 (F2P)
PRE5 (F2P)
Overlay
β1-GFP
NUP100 (F2P)
AVO2 (L2P)
HUL5 (L2P)
FUB1 (I2P)
RPN9 (F2P)
Overlay
β1-GFP

Fig S4: Loss of the non-catalytic subunits of the different N-acetylation complexes results in the same phenotype as loss of the catalytic subunit and N-acetylation status of defined proteins did not affect the localization of the proteasome in starved cells
(A) Loss of NatC activity by knockout of one of its subunits (Mak3, Mak10 and Mak31) or exchange for a catalytic inactive Mak3 leads to an altered proteasome localization after a five day starvation period. (**B**) Cells showing nuclear retention, PSGs or a combination of both were scored in three independent experiments. Approximately 200 cells were scored in two independent samples per condition. Significance was calculated with a non-paired, two-tailed t-test (* = $p<0.05$, ** = $p<0.01$). **(C)** Loss of any single NatA subunit (Nat1, Nat5 and Ard1) does not lead to different proteasome localization after a five day starvation period. (**D**) Quantification as in (**B**). **(E)** Loss of any single NatB subunit (Nat3 and Mdm20) alters proteasome localization after a five day starvation period. (**F**) Quantification as in (**B**). **(G)** Several predicted NatC substrate proteins were N-terminally mutated to test whether their N-acetylation status was important for proteasome localization in starvation. An X2P mutation was made of α6 (Pre5), α5 (Pup2) , Nup100, Avo2, Hul5, FUB1 and RPN9 and these strains were subjected to a five day starvation period. All X2P mutants showed a proteasome localization similar to the WT and can thus be excluded as a NatC substrate influencing proteasome localization. (Scale bars, 5 μm)

Table S1

List of used strains:

**Strain:**	**Genotype:**
NKI4103*	MAT@ can1d::STE2pr-Sp_his5 lyp1d his3d1 leu2d0 ura3d0 met15d0 LYS2+<br>pre3::PRE3-V5-loxP-HA-yEGFP-HYG-loxP-T7-mRFP<br>lyp1d::NATMX-GPD_CRE_EBD78
NKI5537	NKI4103 + *mak3Δ::URA3*
NKI5538	NKI4103 + *mak31Δ::LEU2*
NKI5539	NKI4103 + *mak10Δ::KanMX4*
NKI5540	NKI4103 + *nat3Δ::URA3*
NKI5541	NKI4103 + *mdm20Δ::URA3*
NKI5542	NKI4103 + *ard1Δ::URA3*
NKI5543	NKI4103 + *nat1Δ::URA3*
NKI5544	NKI4103 + *nat5Δ::URA3*
NKI5545	NKI4103 + *mak3Δ::KanMX4, nat3Δ::URA3*
NKI5546	NKI4103 + *mak3*::*MAK3*(N123A,Y130A)
NKI4101*	MAT@ can1d::STE2pr-Sp_his5 lyp1d his3d1 leu2d0 ura3d0 met15d0 LYS2+<br>pre3::PRE3-V5-loxP-HA-yEGFP-HYG-loxP-T7-mRFP
NKI5547	NKI4101 + *pre5*::NatNT2-P_{CYC1}-*PRE5*(F2P)
NKI5548	NKI4101 + *pup2*::NatNT2-P_{CYC1}-*PUP2* (F2P)
NKI5549	NKI4101 + *pre5*::NatNT2-P_{CYC1}-*PRE5*(F2P)<br>*pup2*::KanMX4-P_{CYC1}-*PUP2*(F2P)
NKI5550	NKI4101 + *nup100*::KanMX4-P_{CYC1}-*NUP100*(F2P)
NKI5551	NKI4101 + *avo2*::NatNT2-P_{CYC1}-*AVO2*(L2P)-3xFlag-KanMX4
NKI5552	NKI4101 + *hul5*::NatNT2-P_{CYC1}-*HUL5*(L2P)
NKI5553	NKI4101 + *fub1*::NatNT2-P_{CYC1}-*FUB1*(I2P)
NKI5554	NKI4101 + *rpn9*::NatNT2-P_{CYC1}-*RPN9*(L2P)
NKI4105	MAT@ can1d::STE2pr-Sp_his5 lyp1d his3d1 leu2d0 ura3d0 met15d0 LYS2+<br>pre8::PRE8-V5-loxP-HA-yEGFP-HYG-loxP-T7-mRFP<br>lyp1d::NATMX-GPD_CRE_EBD78
NKI5555	NKI4105 + *ard1Δ::URA3*
NKI5556	NKI4105 + nat3*Δ::URA3*
NKI5557	NKI4105 + *mak3Δ::URA3*
NKI4121	MAT@ can1d::STE2pr-Sp_his5 lyp1d his3d1 leu2d0 ura3d0 met15d0 LYS2+<br>rpn1::RPN1-V5-loxP-HA-yEGFP-HYG-loxP-T7-mRFP<br>lyp1d::NATMX-GPD_CRE_EBD78
NKI5558	NKI4121 + *ard1Δ::URA3*
NKI5559	NKI4121 + nat3*Δ::URA3*
NKI5560	NKI4121 + *mak3Δ::URA3*

* As described in Verzijlbergen *et al* 2009
